# Muscle strengthening activities: cross-sectional associations with skeletal muscle outcomes in adults aged 50–64 and 65 years and above

**DOI:** 10.1007/s41999-025-01327-4

**Published:** 2025-10-13

**Authors:** Konstantinos Prokopidis, Stefano Cacciatore, Paolo Piaggi, Davide Liborio Vetrano, Mathias Schlögl

**Affiliations:** 1 Department of Musculoskeletal Ageing and Science, Institute of Life Course and Medical Sciences, University of Liverpool, 6 West Derby St, Liverpool, L7 8TX UK; 2 Department of Geriatrics, Orthopedics and Rheumatology, Università Cattolica del Sacro Cuore, L.go F. Vito 1, 00168 Rome, Italy; 3 Department of Information Engineering, University of Pisa, Pisa, Italy; 4 Department of Neurobiology, Aging Research Center, Care Sciences and Society, Karolinska Institutet and Stockholm University, Solna, Sweden; 5 Stockholm Gerontology Research Center, Stockholm, Sweden; 6 Department for Geriatric Medicine, Clinic Barmelweid, Erlinsbach, Switzerland

**Keywords:** Sarcopenia, Resistance training, Muscle strength, Gait speed, Muscle mass

## Abstract

**Aim:**

To examine the association of muscle strengthening activities with knee extension strength, gait speed, and skeletal muscle index in adultsaged 50-64 and ≥65 years.

**Findings:**

Muscle strengthening activities are linked to better gait speed, knee extension strength, and skeletal muscle index mainly in middle-aged adults (50-64 years), with weaker or no associations in older adults except for higher activity frequency (≥8 sessions/month), benefi tingstrength in those ≥65 years.

**Message:**

Muscle strengthening activities are linked to better physical function and muscle health in middle-aged compared to older adults,although frequency may be a confounding parameter.

**Background:**

This study examined the association of muscle strengthening activities (MSA) with knee extension strength (KES) and gait speed (GS) (n = 2169), and skeletal muscle index (SMI; n = 765) in adults aged 50–64 and ≥ 65 years.

**Methods:**

Data were drawn from the National Health and Nutrition Examination Survey 1999–2018 cycles. MSA were self-reported based on engagement with weightlifting, push-ups, or sit-ups. MSA frequency was categorized as ≥ 8 or < 8 sessions/month. Linear and logistic regressions were performed, adjusting for demographic and clinical covariates.

**Results:**

MSA were associated with improved GS and KES across adults aged 50–64 years in fully adjusted models (GS: β = -0.24, 95% CI − 0.42 to − 0.07; KES: β = 31.7, 95% CI 18.9 to 44.5) but not in those ≥ 65 years (GS: *p* = 0.07; KES: *p* = 0.11). For SMI, a significant positive association emerged only in the 50–64-year old group after adjustments (β = 0.18, 95% CI 0.03 to 0.34; ≥ 65 years → *p* = 0.53). Age interaction (≥ 65 vs. 50–64 years) showed significant MSA associations with GS and KES, though SMI results were inconsistent. Finally, higher MSA frequency for ≥ 65 versus 50–64 years was linked to higher KES (β = 22.0, *p* = 0.03), but not GS (*p* = 0.05) or SMI (*p* = 0.64).

**Conclusions:**

MSA are associated with higher KES and GS in middle-aged, but not in older adults. Higher MSA frequency is linked to increased KES in older adults.

**Supplementary Information:**

The online version contains supplementary material available at 10.1007/s41999-025-01327-4.

## Introduction

Sarcopenia, the progressive loss of muscle mass and strength [1], is a clinical concern that is more apparent from the fifth decade and is accelerated by each passing decade [2]. Sarcopenia may contribute to the onset of disability, fall risk, and loss of independence, reducing quality of life and increasing risk of mortality [3, 4]. Worldwide, the prevalence of sarcopenia in adults aged 60 years and above is estimated at 10–16% [5], creating a pressing need to identify modifiable factors that could mitigate its adverse effects on muscle function. Among these, muscle strengthening activities (MSA) have received considerable attention for their potential to preserve or enhance muscle mass, strength, and overall function, and may reduce the risk of mortality [6, 7]. The World Health Organisation suggests two or more days/week of MSA engagement of moderate-to-high intensity, such as weightlifting, resistance band use, push-ups, sit-ups, climbing stairs, and/or heavy gardening [8]. In particular, the effects of MSA on key physical performance measures, such as knee extension strength (KES), gait speed (GS), and skeletal muscle index (SMI), are of paramount interest in ageing research for both clinicians and researchers [9, 10].

The benefits of MSA are well documented, as they stimulate muscle hypertrophy, increase muscle strength, and improve functional outcomes in older populations [11]. Studies have shown that MSA can enhance lower-body strength, contributing to better physical performance, including improved GS and KES [7, 12–15]. However, research has also shown that home-based unsupervised exercise, may not be as effective in enhancing physical performance measures in those 65 years and above [16], raising concerns about MSA performance (e.g., lower intensity) and/or physiological mechanisms (e.g., anabolic resistance). Nevertheless, these physical outcomes are crucial in maintaining mobility and preventing falls, both of which are common concerns in older adults. KES and GS, as measures of lower body strength and physical function, respectively, have been linked to functional independence, and deficits in these measures are associated with an increased risk of disability and falls [17, 18]. Furthermore, SMI, which quantifies muscle mass relative to body size, is another important indicator of sarcopenia and functional decline, that can be augmented through MSA [19].

Age is a critical factor in the relationship between MSA and physical function, as the capacity for muscle adaptation declines with advancing age [20]. In this context, while MSA have been shown to counteract the effects of age-related muscle loss, the degree to which they can improve muscle strength and function in older adults is influenced by other age-related factors, including changes in muscle fiber composition, hormone levels, and mitochondrial function [21]. The physiological responses underpinning ageing are contributors to anabolic resistance, where older adults may need to engage with higher dosages of MSA to maintain or improve their hypertrophic and strength outcomes [22]. Therefore, although research has explored the impact of MSA and overall exercise in older adults, whether there are age-specific differences in the perceiving benefits between different age groups in older age could support clinicians in tailoring and prescribing exercise regimes accordingly. Based on above, MSA may confer less muscle health-related benefits in older populations. The aim of this study was to investigate the association between MSA and key sarcopenia outcomes, specifically KES, GS, and SMI, in middle-aged and older adults, to unravel potential associations that may underpin ageing.

## Methods

Data for this study were drawn from multiple cycles of the National Health and Nutrition Examination and Survey (NHANES), a series of cross‐sectional surveys conducted by the National Center for Health Statistics (NCHS) of the US Centers for Disease Control and Prevention (CDC). Specifically, data on KES and GS were obtained from the 1999–2000 and 2001–2002 cycles, while SMI data were derived from the 2001–2002 and 2011–2018 cycles. NHANES collects nationally representative health data through structured interviews, physical examinations, and laboratory assessments. The NHANES recruits participants using a multistage probability sampling design, stratified by region and demographics, then randomly selected, ensuring diversity in the US population. The survey protocols were approved by the NCHS Ethics Review Committee, and all participants provided written informed consent. NHANES data are publicly available via the NCHS website (www.cdc.gov/nchs). The present study adhered to ethical principles outlined in the Declaration of Helsinki and followed the STROBE guidelines for reporting observational research [23]. A flowchart detailing the sample selection is shown in Supplementary Fig. 1.

### General characteristics of the population

The following demographic, clinical, and biochemical variables were considered in the analysis. Demographic information comprised age, sex, self-identified race/ethnicity, and educational attainment. Race was categorized as Mexican American, other Hispanic, non-Hispanic White, non-Hispanic Black, or other. Education was grouped into four levels: college or above, college or associate degree, high school, and below high school. Anthropometric data were collected to calculate body mass index (BMI), derived by dividing measured weight in kilograms by the square of height in meters. Clinical variables were based on self-reported physician diagnoses and included the presence or absence of arthritis, cancer, and diabetes. Biochemical measurements were conducted in accordance with standardized NHANES laboratory protocols. Serum biomarkers analyzed included total cholesterol and high-density lipoprotein (HDL) cholesterol, as indicators of lipid metabolism; serum albumin, as a marker of nutritional and inflammatory status; and C-reactive protein (CRP), a sensitive systemic inflammation marker.

### Muscle strengthening activities

Engagement in MSA was determined through two self-administered questions. Participants were first asked whether they had participated in any exercises aimed at building or maintaining muscular strength in the past 30 days, including examples such as weightlifting, push-ups, or sit-ups. MSA responses were dichotomized: “Yes” (coded as 1), and both “No” and “Unable to perform MSA” (coded as 0). Those who answered affirmatively were then asked to indicate the number of times they performed these activities during that period. Based on the total monthly frequency, participants were classified into two categories: those who performed muscle-strengthening activities eight or more times per month (equivalent to at least twice per week) and those who engaged in them fewer than eight times per month. This cut-off aligns with the frequency recommendations set by the World Health Organization [8].

### Assessment of knee extension strength, gait speed, and skeletal muscle index

Knee extension peak torque was assessed using an isokinetic dynamometer (Kin Com, Chattex Corp., Chattanooga, TN), with maximal voluntary concentric muscle force of the right leg was recorded in Newtons (N) at an angular velocity of 1.05 rad/s (60°/s). Each participant performed submaximal warm-up trials followed by three maximal voluntary effort attempts. The highest value among the three trials was considered the peak force. Peak torque (Nm) was then calculated by multiplying the peak force by the mechanical arm length (in meters), defined as the distance from the ankle to the knee joint.

GS was measured over a 20-foot course at the participant's usual walking pace. A stopwatch was used to record the time from the initial footfall at the starting line to when the participant’s foot crossed the finish line.

SMI was estimated using a validated prediction equation [24]. Body composition was assessed via dual-energy X-ray absorptiometry (DXA) in 765 individuals, including 382 men and 383 women. Lean body mass (excluding bone mineral content) from the upper and lower limbs was summed and normalized by height squared to calculate SMI (kg/m^2^).

### Statistical analysis

Characteristics of the included participants were compared using student’s t-test for continuous variables that were normally distributed. In addition, characteristics of the included participants were compared using the Mann–Whitney U test for continuous variables when they were not normally distributed. Chi-squared test was used for categorical variables. To assess the odds between MSA (Yes/No) (dependent variable) and higher/lower KES, GS, or SMI (independent variables), logistic regression models were used to calculate odds ratios (OR) with 95% confidence intervals (95% CI). High KES, GS, or SMI were classified according to whether values were above the cohort’s 50th percentile. To explore the association of MSA with KES, GS, or SMI as continuous variables, a linear regression was performed. Furthermore, MSA frequency was categorized as ≥ 8 or < 8 sessions/month as a dichotomous variable, for which, further linear and logistic regression analyses were conducted. Finally, an interaction analysis was performed, using age and MSA, to explore the associative interactions between age groups (≥ 65 vs. 50–64 years) for both MSA as “Yes/No” and MSA as “ ≥ 8 or < 8 sessions/month”.

Three models were constructed: Model 1 was unadjusted; Model 2 adjusted for age, sex, BMI, race, and education; and Model 3 additionally adjusted for the presence of arthritis, diabetes, and cancer. All analyses were also stratified by age groups: 50–64 years and ≥ 65 years. A two-tailed *p*-value of 0.05 was considered statistically significant. Statistical analyses were performed using IBM SPSS Statistics, v29.0.

## Results

### Sample characteristics for knee extension strength and gait speed analyses

A total of 2169 adults aged 50 years or older were included in the analysis of KES and GS, comprising 1126 participants aged 50–64 years and 1043 participants aged ≥ 65 years. The sample size was based on filtering for available data regarding age, BMI, comorbidities, as well as KES, GS, or SMI (Supplementary Fig. 1). The overall mean age was 64.9 ± 9.9 years, with younger and older subgroups averaging 56.9 ± 4.5 and 73.6 ± 6.1 years, respectively. The sex distribution was balanced across age groups, with males and females each representing approximately 50% of the sample (50–64 years: 50.1% males; ≥ 65 years: 50.9% males; *p* = 0.70). BMI was higher in the younger group (28.8 ± 5.5 kg/m^2^) compared to older adults (27.3 ± 4.8 kg/m^2^, *p* < 0.01). Total cholesterol was slightly elevated in the 50–64 age group (205.4 ± 60.8 mg/dL vs. 199.7 ± 62.2 mg/dL, *p* = 0.03), while serum albumin was also modestly higher (4.11 ± 0.93 g/L vs. 4.02 ± 0.99 g/L, *p* = 0.03). No significant differences were observed for HDL cholesterol (50.1 ± 19.2 vs. 50.7 ± 20.7 mg/dL, *p* = 0.46) or CRP levels (4.39 ± 6.72 vs. 4.55 ± 8.25 mg/L, *p* = 0.61). The prevalence of arthritis and cancer was higher among participants aged ≥ 65 years (36.5% and 17.7%, respectively) compared to those aged 50–64 years (30.0% and 10.0%, both *p* < 0.01), while diabetes prevalence did not differ significantly between groups (*p* = 0.61). In terms of functional measures, GS was slower among older adults (6.90 ± 2.61 s) than younger counterparts (5.80 ± 1.30 s, *p* < 0.01), and KES was lower in the older group (305.4 ± 102.2 Nm vs. 393.5 ± 123.2 Nm, *p* < 0.01). Comprehensive details for each groups’ characteristics are presented in Tables 1 and 2.

**Table 1:** Baseline characteristics of the cohort, including data on gait speed and knee extension. Data is expressed as mean (standard deviation)

	All (n = 2169)	Aged 50–64 (n = 1126)	Aged ≥ 65 (n = 1043)	*p*-value
Age (years)	64.9 (9.9)	56.9 (4.5)	73.6 (6.1)	< 0.01*
Sex (males/females)	1095/1074	564/562	531/512	0.70
Race (%)				
Mexican American	17.8	19.5	16.0	
Other Hispanic	3.9	4.7	3.1	0.46
Non-Hispanic White	59.0	54.6	63.7	
Non-Hispanic Black	16.4	17.5	15.2	
Other Race	2.9	3.7	2.0	
Education (%)				
College or above	21.3	26.1	16.2	
College or associate degree	22.7	24.3	21.0	< 0.01*
High school	22.3	20.4	24.3	
Other (below high school)	33.7	29.2	38.5	
Body mass index (kg/m^2^)	28.1 (5.3)	28.8 (5.5)	27.3 (4.8)	< 0.01*
Arthritis (%)	33.1	30.0	36.5	< 0.01*
Cancer (%)	13.7	10.0	17.7	< 0.01*
Diabetes (%)	14.9	13.1	16.8	0.61
Total cholesterol (mg/dL)	202.6 (61.5)	205.4 (60.8)	199.7 (62.2)	0.03*
High-density lipoprotein cholesterol (mg/dL)	50.4 (20.0)	50.1 (19.2)	50.7 (20.7)	0.46
C-reactive protein (mg/L)	4.47 (7.50)	4.39 (6.72)	4.55 (8.25)	0.61
Serum albumin (g/L)	4.06 (0.96)	4.11 (0.93)	4.02 (0.99)	0.03*
Gait speed (seconds/20 ft)	6.33 (2.11)	5.80 (1.30)	6.90 (2.61)	< 0.01*
Knee extension (Nm)	351.2 (121.8)	393.5 (123.2)	305.4 (102.2)	< 0.01*

^*^Indicates significance at *p* < 0.05

Gait speed was normally distributed

**Table 2:** Baseline characteristics of the cohort, including primary data on skeletal muscle index. Data is expressed as mean (standard deviation)

Outcomes	All (n = 765)	Aged 50–64 (n = 418)	Aged ≥ 65 (n = 347)	*p*-value
Age (years)	64.3 (9.5)	57.1 (4.4)	72.9 (6.1)	< 0.01*
Sex (males/females)	382/383	203/215	179/168	0.41
Race (%)				
Mexican American	22.5	24.9	19.6	
Other Hispanic	5.4	6.7	3.7	0.29
Non-Hispanic White	52.5	47.4	59.4	
Non-Hispanic Black	16.2	17.7	14.4	
Other Race	3.4	3.9	2.9	
Education (%)				
College or above	16.7	21.8	10.7	
College or associate degree	20.3	23.0	17.0	< 0.01*
High school	22.7	18.9	27.4	
Other (below high school)	40.3	36.3	44.9	
Body mass index (kg/m^2^)	28.1 (5.3)	28.8 (5.4)	27.4 (5.0)	< 0.01*
Arthritis (%)	26.7	28.5	24.5	0.22
Cancer (%)	10.3	10.0	10.9	0.78
Diabetes (%)	13.2	11.2	15.6	0.29
Total cholesterol (mg/dL)	202.1 (63.7)	205.3 (59.7)	198.1 (68.1)	0.12
High-density lipoprotein cholesterol (mg/dL)	48.9 (19.9)	49.3 (19.0)	48.4 (21.0)	0.53
C-reactive protein (mg/L)	4.66 (7.17)	4.52 (6.70)	4.83 (7.72)	< 0.01*
Serum albumin (g/L)	4.13 (1.06)	4.22 (0.94)	4.02 (1.17)	0.53
Gait speed (seconds/20 ft)	6.52 (2.68)	5.96 (1.34)	7.20 (3.58)	< 0.01*
Knee extension (Nm)	353.1 (124.1)	390.7 (125.1)	307.9 (106.8)	< 0.01*
Skeletal muscle index (kg/m^2^)	7.36 (1.43)	7.59 (1.45)	7.09 (1.35)	< 0.01*

^*^Indicates significance at *p* < 0.05

Gait speed and skeletal muscle index were normally distributed

### Results from interaction analyses (≥ 65 vs. 50–64 years)

Our findings showed a significant association in performing versus not performing MSA with both GS and KES in the unadjusted (GS → β = − 0.29, 95%CI − 0.44 to − 0.14, *p* = 0.03; KES → β = 10.7, 95%CI 1.9–19.4, *p* = 0.03) and fully adjusted models (GS → β = − 0.17, 95%CI − 0.32 to − 0.02, *p* = 0.02; KES → β = 7.9, 95%CI 1.0–14.8, *p* = 0.02) (Table 3). Similar outcomes were shown when a logistic regression was used for low GS (Model 3: OR = 2.94, 95%CI 2.43–3.56, *p* < 0.01) and high KES (Model 3: OR = 0.22, 95%CI 0.17–0.28, *p* < 0.01) (Table 3). Similar results were yielded in relation to SMI as a continuous variable (Model 3: β = − 0.34, 95%CI − 0.42 to − 0.25, *p* < 0.01), but not when high SMI was used categorically (Model 3: OR = 1.17, 95%CI 0.87–1.56, *p* = 0.31) (Table 4). When MSA frequency (≥ 8 or < 8 sessions/month) was applied to test the association with GS, although significant link was found in the unadjusted model (β = − 0.14, 95%CI − 0.22 to − 0.06, *p* < 0.01), no statistical association was found following all confounders in model 3 (β = − 0.08, 95%CI − 0.16 to − 0.08, *p* = 0.05) (Table 5). A significant association was found with KES across models (Model 3: β = 4.2, 95%CI 0.5 to 7.9, *p* = 0.03), but not with SMI (Model 3: β = 0.01, 95%CI − 0.04 to 0.06, *p* = 0.64) (Table 5).

**Table 3:** Association of performing muscle strengthening activities with (increased) knee extension strength and (slower) gait speed in adults 50 years of age and above

	**Model 1**			**Model 2**			**Model 3**		
*Aged ≥ 50 years*									
	***p***	**b**	**95%CI**	***p***	**b**	**95%CI**	***p***	**b**	**95%CI**
Gait speed (sec)	< 0.01*	− 0.67	− 0.90 to − 0.44	< 0.01*	− 0.32	− 0.54 to − 0.11	< 0.01*	− 0.31	− 0.53 to − 0.09
Knee extension (Nm)	< 0.01*	39.5	26.4–52.7	< 0.01*	23.7	14.5–32.9	< 0.01*	23.3	14.1–32.5
	***p***	**OR**	**95%CI**	***p***	**OR**	**95%CI**	***p***	**OR**	**95%CI**
Low gait speed	< 0.01*	0.47	0.37–0.58	< 0.01*	0.64	0.50–0.83	< 0.01*	0.65	0.50–0.83
High knee extension strength	< 0.01*	1.69	1.35–2.11	< 0.01*	1.66	1.24–2.24	< 0.01*	1.65	1.23–2.23

*Aged 50–64 years*									
	***p***	**b**	**95%CI**	***p***	**b**	**95%CI**	***p***	**b**	**95%CI**
Gait speed (sec)	< 0.01*	− 0.52	− 0.71 to − 0.33	< 0.01*	− 0.25	− 0.42 to − 0.07	< 0.01*	− 0.24	− 0.42 to − 0.07
Knee extension (Nm)	< 0.01*	42.4	24.8 to 60.0	< 0.01*	32.3	19.4 to 45.2	< 0.01*	31.7	18.9 to 44.5
	***p***	**OR**	**95%CI**	***p***	**OR**	**95%CI**	***p***	**OR**	**95%CI**
Low gait speed	< 0.01*	0.44	0.32–0.61	< 0.01*	0.61	0.43–0.86	< 0.01*	0.61	0.43–0.87
High knee extension strength	< 0.01*	1.79	1.30–2.47	< 0.01*	1.94	1.29–2.92	< 0.01*	1.92	1.27–2.89

*Aged ≥ 65 years*									
	***p***	**b**	**95%CI**	***p***	**b**	**95%CI**	***p***	**b**	**95%CI**
Gait speed (sec)	< 0.01*	− 0.66	− 1.10 to − 0.23	0.06	− 0.41	− 0.83 to 0.01	0.07	− 0.39	− 0.80 to 0.03
Knee extension (Nm)	0.03*	18.9	1.94–36.0	0.10	10.9	− 1.9 to 23.7	0.11	10.4	− 2.4 to 23.3
	***p***	**OR**	**95%CI**	***p***	**OR**	**95%CI**	***p***	**OR**	**95%CI**
Low gait speed	< 0.01*	0.54	0.38–0.75	0.04*	0.69	0.48–0.99	0.045*	0.69	0.48–0.99
High knee extension strength	0.05	1.40	1.00–1.97	0.20	1.33	0.86–1.06	0.22	1.32	0.85–2.04

*Age interaction (≥ 65 vs. 50–64 years)*									
	***p***	**b**	**95%CI**	***p***	**b**	**95%CI**	***p***	**b**	**95%CI**
Gait speed (sec)	< 0.01*	− 0.29	− 0.44 to − 0.14	0.03*	− 0.17	− 0.32 to − 0.02	0.02*	− 0.17	− 0.32 to − 0.02
Knee extension (Nm)	0.02*	10.7	1.9–19.4	0.03*	7.5	0.6–14.4	0.02*	7.9	1.0–14.8
	***p***	**OR**	**95%CI**	***p***	**OR**	**95%CI**	***p***	**OR**	**95%CI**
Low gait speed	< 0.01*	3.00	2.52–3.57	< 0.01*	2.99	2.47–3.60	< 0.01*	2.94	2.43–3.56
High knee extension strength	< 0.01*	0.31	0.26–0.37	< 0.01*	0.21	0.17–0.27	< 0.01*	0.22	0.17–0.28

^*^Indicates significance at *p* < 0.05

Model 1: unadjusted

Model 2: adjusted for age, sex, body mass index, race, and education

Model 3: adjusted for Model 2 and arthritis, cancer, and diabetes

**Table 4:** Association of performing muscle strengthening activities with (higher) skeletal muscle index in adults 50 years of age and above

	**Model 1**			**Model 2**			**Model 3**		
*Aged ≥ 50 years*									
	***p***	**b**	**95%CI**	***p***	**b**	**95%CI**	***p***	**b**	**95%CI**
Skeletal muscle index (kg/m^2^)	0.96	0.007	− 0.28 to 0.29	0.05	0.118	− 0.00 to 0.24	0.04*	0.123	0.00–0.24
	***p***	**OR**	**95%CI**	***p***	**OR**	**95%CI**	***p***	**OR**	**95%CI**
High Skeletal muscle index (kg/m^2^)	0.67	0.92	0.62–1.37	0.76	1.12	0.55–2.28	0.67	1.17	0.57–2.41

*Aged 50–64 years*									
	***p***	**b**	**95%CI**	***p***	**b**	**95%CI**	***p***	**b**	**95%CI**
Skeletal muscle index (kg/m^2^)	0.19	− 0.252	− 0.63 to 0.12	0.03*	0.176	0.02–0.033	0.02*	0.183	0.03–0.34
	***p***	**OR**	**95%CI**	***p***	**OR**	**95%CI**	***p***	**OR**	**95%CI**
High Skeletal muscle index (kg/m^2^)	0.14	0.68	0.40–1.13	0.54	1.35	0.52–3.50	0.43	1.48	0.56–3.94

*Aged ≥ 65 years*									
	***p***	**b**	**95%CI**	***p***	**b**	**95%CI**	***p***	**b**	**95%CI**
Skeletal muscle index (kg/m^2^)	0.17	0.299	− 0.13 to 0.73	0.64	0.044	− 0.14 to 0.23	0.53	0.060	− 0.13 to 0.25
	***p***	**OR**	**95%CI**	***p***	**OR**	**95%CI**	***p***	**OR**	**95%CI**
High Skeletal muscle index (kg/m^2^)	0.38	1.33	0.71–2.51	0.76	0.83	0.27–2.62	0.76	0.83	026–2.66

*Age interaction (≥ 65 vs. 50–64 years)*									
	***p***	**b**	**95%CI**	***p***	**b**	**95%CI**	***p***	**b**	**95%CI**
Skeletal muscle index (kg/m^2^)	< 0.01*	− 0.50	− 0.70 to − 0.30	< 0.01*	− 0.33	− 0.42 to − 0.25	< 0.01*	− 0.34	− 0.42 to − 0.25
	***p***	**OR**	**95%CI**	***p***	**OR**	**95%CI**	***p***	**OR**	**95%CI**
High Skeletal muscle index (kg/m^2^)	0.29	1.17	0.88–1.55	0.32	1.16	0.87–1.56	0.31	1.17	0.87–1.56

^*^Indicates significance at *p* < 0.05

Model 1: unadjusted

Model 2: adjusted for age, sex, body mass index, race, and education

Model 3: adjusted for Model 2 and arthritis, cancer, and diabetes

**Table 5:** Association of muscle strengthening activity frequency (8 or more vs. 1–7 sessions per month) with knee extension strength, gait speed, and skeletal muscle index in adults 50 years of age and above

	Model 1			Model 2			Model 3		
	*p*	b	95%CI	*p*	b	95%CI	*p*	b	95%CI
Aged ≥ 50 years (n = 397)									
Gait speed (sec)	0.10	0.30	− 0.06 to 0.61	0.42	0.12	− 0.17 to 0.41	0.43	0.12	− 0.18 to 0.41
Knee extension (Nm)	0.57	8.6	− 21.5 to 38.8	0.05	20.3	0.03–40.5	0.03*	22.0	1.7–42.2
Aged ≥ 50 years (n = 114)									
Skeletal muscle index (kg/m^2^)	0.39	− 0.24	− 0.81 to 0.32	0.92	− 0.01	− 0.27 to 0.24	0.85	− 0.02	− 0.28 to 0.23
Aged 50–64 years (n = 164)									
Gait speed (sec)	0.79	0.04	− 0.27 to 0.36	0.64	0.07	− 0.23 to 0.37	0.65	0.07	− 0.23 to 0.37
Knee extension (Nm)	0.03*	40.4	3.6–77.2	0.23	16.6	− 10.5 to 43.7	0.18	18.5	− 8.4 to 45.5
Aged 50–64 years (n = 44)									
Skeletal muscle index (kg/m^2^)	0.21	0.43	− 0.25 to 1.11	0.66	0.07	− 0.25 to 0.39	0.81	0.04	− 0.28 to 0.36
Aged ≥ 65 years (n = 233)									
Gait speed (sec)	0.28	0.36	− 0.30 to 1.02	0.73	− 2.82	− 6.48 to 0.85	0.76	0.10	− 0.51 to 0.70
Knee extension (Nm)	0.57	− 11.9	− 52.8 to 29.0	0.29	16.2	− 13.9 to 46.3	0.28	16.8	− 13.6 to 47.2
Aged ≥ 65 years (n = 70)									
Skeletal muscle index (kg/m^2^)	< 0.01*	− 1.31	− 2.23 to − 0.39	0.81	− 0.06	− 0.54 to 0.42	0.86	0.04	− 0.54 to 0.45
Age interaction (≥ 65 vs. 50–64 years)									
Gait speed (sec)	< 0.01*	− 0.14	− 0.22 to − 0.06	0.06	− 0.08	− 0.16 to 0.003	0.05	− 0.08	− 0.16 to 0.001
Knee extension (Nm)	0.02*	5.5	0.8–10.2	0.04*	3.9	0.1–7.6	0.03*	4.2	0.5–7.9
Skeletal muscle index (kg/m^2^)	0.66	− 0.02	− 0.13 to 0.08	0.69	0.01	− 0.04 to 0.06	0.64	0.01	− 0.04 to 0.06

^*^Indicates significance at *p* < 0.05

Model 1: unadjusted

Model 2: adjusted for age, sex, body mass index, race, and education

Model 3: adjusted for Model 2 and arthritis, cancer, and diabetes

### Association of muscle strengthening activities with knee extension strength and gait speed

In unadjusted linear regression models (Model 1), performing versus not performing MSA was significantly associated with lower GS time (i.e., faster GS) and higher KES across all age groups. For GS: β = − 0.67 (95% CI − 0.90 to − 0.44, *p* < 0.01) in the full sample; − 0.52 (95% CI − 0.71 to − 0.33, *p* < 0.01) in adults aged 50–64; and − 0.66 (95% CI − 1.10 to − 0.23, *p* < 0.01) in those ≥ 65 years. For KES: β = 39.6 (95%CI 26.4 to 52.7, *p* < 0.01) in the full sample; 42.4 (95%CI 24.8 to 60.0, *p* < 0.01) in adults aged 50–64; and 18.9 (95%CI 1.94 to 36.0, *p* < 0.01) in those ≥ 65 years. However, in adjusted models (Model 2 and Model 3), these associations were no longer statistically significant among adults aged ≥ 65 years. Specifically, for GS: *p* = 0.06 in Model 2 and *p* = 0.07 in Model 3; for KES: *p* = 0.10 in Model 2 and *p* = 0.11 in Model 3. In contrast, significant associations remained in the 50–64 age group after full adjustment (Model 3): GS (β = − 0.24, 95%CI − 0.42 to − 0.07, *p* < 0.01) and KES (β = 31.7, 95%CI 18.9 to 44.5, *p* < 0.01) (Table 3).

Logistic regression analyses further supported these findings. Among adults aged 50–64 years, performing MSA was associated with lower odds of having low GS (OR = 0.44, 95%CI 0.32 to 0.61, *p* < 0.01) and higher odds of high KES (OR = 1.79, 95%CI 1.30 to 2.47, *p* < 0.01) in the unadjusted model. In adults over 65 years, the association with high KES was borderline (*p* = 0.05), and not significant after adjustment (Model 2: *p* = 0.20; Model 3: *p* = 0.22). After full adjustment, significant associations persisted only in the 50–64 age group (low GS: OR = 0.61, 95%CI 0.43 to 0.87, *p* < 0.01; high KES: OR = 1.92, 95%CI 1.27 to 2.89, *p* < 0.01). In contrast, among adults aged 65 years or older, only the association with low GS remained significant (OR = 0.69, 95%CI 0.48 to 0.99, *p* = 0.045), while the association with high KES was not (Table 3).

### Association of muscle strengthening activities with skeletal muscle index

Linear regression analyses showed no significant association between MSA engagement and SMI in the unadjusted model (Model 1) for the overall sample (*p* = 0.96) or when stratified by age group (50–64 years: *p* = 0.19; ≥ 65 years: *p* = 0.17). In adjusted analyses, a significant positive association between MSA engagement and SMI emerged only in adults aged 50–64 years. In this age group, Model 2 showed a β of 0.176 (95%CI 0.02 to 0.33, *p* = 0.03), which remained significant in Model 3 (β = 0.183, 95%CI 0.03 to 0.34, *p* = 0.02). Conversely, no significant associations were observed among adults aged 65 years or older in either adjusted model (Model 2: *p* = 0.64; Model 3: *p* = 0.53). In the total sample, the fully adjusted model (Model 3) yielded a borderline significant association (β = 0.123, 95%CI 0.00 to 0.24, *p* = 0.04) (Table 4).

Results from binary logistic regression did not show significant associations between MSA engagement and having high SMI in any age group or model. In the fully adjusted model (Model 3), the odds ratio for high SMI in relation to MSA was 1.17 (*p* = 0.67) in the total sample, 1.48 (*p* = 0.43) in adults aged 50–64, and 0.83 (*p* = 0.76) in those aged 65 years or older (Table 4).

### Association between frequency of muscle-strengthening activities and physical outcomes

Analyses exploring the frequency of muscle-strengthening activities (≥ 8 sessions/month vs. 1–7 sessions/month) showed limited and inconsistent associations with muscle outcomes (Table 5). In the overall sample (aged ≥ 50 years), higher activity frequency was associated with greater KES in the fully adjusted model (β = 22.0, 95%CI 1.7 to 42.2, *p* = 0.03), whereas no significant association was observed for GS in any model. When stratified by age, this positive association with KES was seen only in the 50–64 age group in the unadjusted model (β = 40.4, 95%CI 3.6 to 77.2, *p* = 0.03), but the association was attenuated and no longer significant after adjustment. Among adults aged ≥ 65 years, no significant associations were observed for either GS or KES.

Regarding SMI, no significant associations were found in any age group or model (Table 5). Notably, in the unadjusted model for adults aged ≥ 65 years, higher frequency of MSA was associated with lower SMI (β = − 1.31, 95%CI − 2.23 to − 0.39, *p* < 0.01), though this association disappeared after adjusting for covariates.

## Discussion

This study provides important insights into the association between MSA and key muscle-related outcomes, including KES, GS, and SMI, in middle-aged and older adults. Notably, age-related differences (≥ 65 vs. 50–64 years) significantly influenced the association between MSA and both GS and KES, with consistent effects observed in adjusted models, though this association with SMI and MSA frequency was omitted. In addition, consistent associations were observed only among adults aged 50–64 years, while attenuated or non-significant associations were found in those aged 65 years or older following full adjustment for covariates. Understanding associative parameters of muscle mass and strength is critical for exploring mediating factors that may support maintaining overall health, independence, and quality of life in older adults.

The consistent association between KES, GS, and MSA in the multivariate model across only the 50–64-year age group may have several explanations. Reverse causation cannot be excluded, as older adults may already have lower muscle mass and strength, along with more comorbidities, which may limit their ability to perform MSA at comparable frequency or intensity. Additionally, the lower training volume or progressive overload in older adults may contribute to the diminished response. Since MSA engagement was based on self-reported questionnaires, it is plausible that older participants did not engage in exercise with sufficient intensity or frequency to elicit significant adaptations. Both exercise intensity and volume are critical to consider when implementing resistance exercise-based interventions, particularly when targeting muscular strength in older populations [25, 26]. Furthermore, older adults may experience increased daily fatigue or encounter environmental and psychosocial barriers such as lack of professional supervision and social/family support, or fear of injury, which can reduce adherence and effectiveness of MSA programs [27, 28]. This is critical to consider as engagement with MSA should be a continuous process and any targeted interventions should promote higher adherence to exercise programmes in order to sustain results aligning with better muscle-related responses [29].

From a physiological standpoint, anabolic resistance and age-related impairments in neuromuscular adaptation may also contribute to the diminished response to MSA in older adults. Previous studies have shown that reductions in type II muscle fibers and their contractile proteins (e.g., myosin heavy chain IIa and IIx) are more pronounced in adults over 65 years, potentially blunting muscular gains [30]. Nonetheless, resistance training remains effective for improving muscle strength across age groups [31], although the degree of adaptation may vary.

Interestingly, MSA was associated with higher strength and physical function but not with SMI, particularly in older adults. These findings reinforce the current understanding that sarcopenia is not solely defined by muscle mass loss [32]. While Rosenberg initially described sarcopenia as the age-related decline in skeletal muscle mass, contemporary definitions emphasize low muscle strength and poor physical performance as the key diagnostic criteria [33]. Therefore, the lack of association between MSA and SMI may reflect the functional focus of resistance training, which often leads to neuromuscular improvements without necessarily increasing muscle mass, especially in older adults who may be subject to anabolic resistance. These findings also have relevant public health and clinical implications. First, they highlight the importance of promoting muscle health beginning in midlife, before the steep decline in functional capacity typically observed in later years. Tailoring MSA guidelines according to age and functional status may improve engagement and efficacy. Moreover, the promotion of supervised, progressive resistance training programs, particularly in adults over 50, could help mitigate sarcopenia onset and progression, potentially preserving independence and reducing fall risk [34].

It is worth noting that our cohort included adults of up to 85 years of age. However, a more granular analysis across narrower age bands (e.g., ≥ 70, ≥ 75 years) was not feasible due to limited statistical power. This limitation may have obscured potentially meaningful subgroup-specific trends.

### Clinical implications

The present findings underscore the importance of implementing MSA as a core component of clinical and public health strategies to preserve muscle function across ageing. Current recommendations advocate resistance training at least twice per week, targeting major muscle groups with moderate intensity (55–80% of one-repetition maximum) and 1–3 sets of 8–12 repetitions, progressing to volitional fatigue. For older adults, particularly those with frailty or chronic conditions, supervised programs are advised to ensure safety and proper technique, and to optimize adherence [35, 36]. While the same principles apply across middle-aged and older adults, those > 65 years may require lower starting intensities, slower progression, and a greater emphasis on balance and functional movements to mitigate fall risk [37]. Additionally, adherence may improve with minimal-dose resistance training approaches (short, frequent sessions), use of accessible equipment, and integration of resistance exercise into daily routines (“exercise snacking”) [38]. Clinicians should prescribe muscle-strengthening exercise at least twice per week for all adults aged ≥ 50 years, starting at low-to-moderate intensity and progressing gradually as tolerated [34]. Importantly, promoting equitable access to health-enhancing physical activity through healthcare infrastructures and community resources may represent a cost-effective strategy to prevent functional decline and foster healthy ageing [39, 40].

### Strengths and limitations

This study attempted to find links between MSA and lower muscle strength and function, and appendicular lean mass in age groups representing the onset and the acceleration of sarcopenia. A major strength lies in the use of a large, nationally representative dataset with standardized measurements and rigorous adjustment for relevant demographic, clinical, and biochemical covariates. Additionally, the statistical analyses employed a variety of covariates pertinent to muscle health with a low variance inflation factor (VIF; < 2) across multiple models.

However, there are limitations that should be acknowledged. The cross-sectional design limits the ability to establish causality, and the reliance on self-reported data for MSA and comorbidities may introduce recall or classification bias. Another limitation concerns the use of the 50th percentile to classify GS, KES, and SMI. Although this method does not correspond to clinically validated thresholds, it offers a simple and reproducible way to distinguish between lower and higher values within the sample. This approach is commonly used in exploratory studies and aligns with the continuous treatment of these variables in linear regression models. Nonetheless, the absence of clinically meaningful cut-offs may limit the interpretability of the results in terms of identifying individuals at risk of sarcopenia, and future studies may consider adopting more conservative thresholds to enhance clinical relevance. The assessment of MSA did not account for intensity, type, duration, or supervision, which are important mediators of exercise effectiveness, particularly in older populations. Furthermore, the sample size for the SMI subgroup was relatively small, reducing statistical power. The lack of data on upper-body strength (e.g., handgrip strength) also limits the comprehensiveness of the analysis, as handgrip is a widely used clinical marker of sarcopenia. Moreover, KES and GS data were gathered from a different cohort compared to adults with data pertinent to SMI, which does not allow for conclusions that may associate these three variables together. Finally, subgroup analyses by narrower age bands (e.g., ≥ 70, ≥ 75, or ≥ 80 years) were not feasible due to insufficient sample size in these strata.

## Conclusion

In conclusion, an association between MSA and both GS and KES in older adults (≥ 65 years) compared to those aged 50–64 years, with significant effects persisting across adjusted models was observed, while associations with SMI and MSA frequency were less consistent. MSA were associated with higher KES and faster GS, primarily among adults aged 50–64 years. These associations were attenuated or were no longer significant in adults aged 65 years and above after adjustments. The lack of consistent associations with SMI was also highlighted in these age groups, separately. These findings support the prescription of muscle-strengthening activities at least twice per week for adults aged 50 years and older, with tailored programs and system-level efforts to facilitate equitable access to health-enhancing physical activity. Future research should further investigate the physiological and behavioural differences in response to resistance exercise across age groups through longitudinal and interventional studies.

## Supplementary Information

Below is the link to the electronic supplementary material.

Supplementary file1 (DOCX 33 KB)

## Data Availability

Data is available upon request.
