# Supplementary material for: Muscle strengthening activities: cross-sectional associations with skeletal muscle outcomes in adults aged 50–64 and 65 years and above
Source: Eur Geriatr Med. 2025 Oct 13;17(1):63–73. doi: 10.1007/s41999-025-01327-4 (PMC12946235; doi:10.1007/s41999-025-01327-4)
Supplement: Supplementary file 1 — Supplementary file1 (DOCX 33 KB) [file 41999_2025_1327_MOESM1_ESM.docx]

**Supplementary Figure 1.** Flowchart of the selected sample size.

**Total**

(*n* = 128,809)

| **Step 1: Filtering for those aged ≥50 years and those with available data on Muscle Strengthening Activities** (*n* = 9633) |
| --- |

**Step 2: Filtering for those with available data based on BMI, Arthritis, Cancer, and Diabetes**
(*n* = 7367)

**Step 3: Filtering for available data on Skeletal Muscle Index**
(*n* = 765)

**Step 3: Filtering for available data on Gait Speed and Knee Extension**

(n = 2169)
